# Separation of Magnesium and Lithium Ions Utilizing Layer-by-Layer Polyelectrolyte Modification of Polyacrylonitrile Hollow Fiber Porous Membranes

**DOI:** 10.3390/ma17235878

**Published:** 2024-11-30

**Authors:** Danai Koukoufilippou, Ioannis L. Liakos, George I. Pilatos, Niki Plakantonaki, Alexandros Banis, Nikolaos K. Kanellopoulos

**Affiliations:** 1 Institute of Nanoscience and Nanotechnology, National Centre for Scientific Research Demokritos, Patr. Gregoriou E & 27 Neapoleos Street, 15341 Agia Paraskevi, Greece; danaikoukoufilippou@gmail.com (D.K.); g.pilatos@inn.demokritos.gr (G.I.P.); n.plakantonaki@inn.demokritos.gr (N.P.); a.banis@inn.demokritos.gr (A.B.); 2 High Technology Filters (HTF) S.A., Siokou Street 18, 15341 Agia Paraskevi, Greece

**Keywords:** Mg^2+^/Li^+^ separation, nanofiltration, layer-by-layer deposition, PAN hollow fiber, polyelectrolytes

## Abstract

This study explores the layer-by-layer (LBL) modification of polyacrylonitrile (PAN) hollow fibers for effective Mg^2+^/Li^+^ separation. It employs an LBL method of surface modification using polyelectrolytes, specifically aiming to enhance ion selectivity and improve the efficiency of lithium extraction from brines or lithium battery wastes, which is critical for battery recycling and other industrial applications. The modification process involves coating the hydrolyzed PAN fibers with alternating layers of positively charged polyelectrolytes, such as poly(allylamine hydrochloride) (PAH), polyethyleneimine (PEI), or poly(diallyldimethylammonium chloride) (PDADMAC) and negatively charged polyelectrolytes, such as poly(styrene sulfonate) (PSS), to form polyelectrolyte multilayers (PEMs). This study evaluates the modified membranes in Mg^2+^ and Li^+^ salt solutions, demonstrating significant improvements in selectivity for Mg^2+^/Li^+^ separation. PAH was identified as the optimal positively charged polyelectrolyte. PAN hollow fibers modified with ten bilayers of PAH/PSS achieved rejection rates of 95.4% for Mg^2+^ ions and 34.8% for Li^+^ ions, and a permeance of 0.39 LMH/bar. This highlights the potential of LBL techniques for effectively addressing the challenges of ion separation across a variety of applications.

## 1. Introduction

The demand for lithium has been rising quickly in recent years, mostly due to the development of lithium-ion batteries [1,2]. Lithium is used extensively in portable electronic devices, the nuclear industry, new energy vehicles, as well as in the ceramics and glass industries [3,4]. A global shortage of Li resources, caused by the rising demand for Li^+^ batteries, has been observed, as industry consumed more than 30% of Li^+^ resources and was predicted to reach 66% by 2025. An estimated 60% of the world’s lithium resources are found in seawater and salt-lake brines, where Li^+^ coexists with other ions like Mg^2+^, K^+^, Ca^2+^ and Na^+^ [1,5]. Lithium extraction from salt-lake brines constitutes a significant challenge, as it requires the effective separation of Mg^2+^ and Li^+^ with comparable hydration radius (0.43 nm and 0.38 nm) [3]. Several techniques have been investigated for lithium extraction and the selective separation of lithium from magnesium, such as chemical precipitation, adsorption, solvent extraction, concentration–evaporation, electrochemical methods, and membrane separation, as well as reaction-coupled separation technology [6,7]. Among these, membrane separation techniques are particularly advantageous due to their low energy consumption and high separation efficiency. The major benefit of membrane processes is their ability to continuously operate with high selectivity, which is essential for handling the similar ionic radius of Mg^2+^ and Li^+^, making them promising for industrial-scale applications. Additionally, membrane separation minimizes the need for chemical additives, which can further reduce operational costs. while maintaining high purity levels for lithium extraction [7]. Specifically, nanofiltration technology, considering its high separation efficiency, low energy consumption [8], easy operational procedures, and environmental friendliness [5,9] is not only an effective method for Li^+^ extraction from natural sources, but is also a promising technology for potential lithium-ion battery recycling. Recycling remains an additional challenge, but also an important process. According to research, 100% of LIBs need to be recycled, with a minimum 90% recovery of lithium, in order to have enough lithium carbonate equivalent for the whole 21st century [10]. Conventional nanofiltration membranes, due to their negatively charged surface, are ineffective for Mg^2+^/Li^+^ separation and Mg^2+^ rejection. Many researchers have explored possible modification methods to improve Mg^2+^/Li^+^ selectivity by producing membranes with positive active layers [11]. A promising approach involves interfacial polymerization utilizing positively charged amine monomers. Membranes produced using this method, incorporating active layers of PEI, branched polyethyleneimine (BPEI), or 1,4-bis(3-aminopropyl)piperazine (BAPP), as well as those modified with additional positively charged components and nanoparticles, demonstrated heightened selectivity (with a general selectivity factor (SF) > 10) [12]. The LBL assembly technique offers a straightforward and environmentally friendly alternative for the surface modification of nanofiltration membranes. This method is based on the formation of separative polyelectrolyte multilayers (PEMs) with an adjustable thickness, achieved through the exposure of their surface to either cationic or anionic polyelectrolytes (PEs) [13]. In the context of Li^+^ ions separation using polyelectrolyte multilayer hollow fibers, the Donnan effect can play a significant role. Donnan exclusion results from electrostatic interaction between ions and the fixed charges on the membrane, predicting a dependence of rejection on the valence type of the electrolyte, with a characteristic increase as the co-ion charge increases [14]. These PEM hollow fibers can act as selective barriers for ion transport based on size, charge, and other physicochemical properties of the ions. LBL nanofiltration membranes have found applications in various fields, including wastewater treatment for micropollutant removal, phosphorus and scandium recovery [12,13], drug delivery, antibacterial or anti-inflammatory coating for implants [15], oil/water separation [13] and Mg^2+^/Li^+^ separation [12]. Numerous research articles in the field of ion separation have demonstrated the efficacy of nanofiltration (NF) membranes modified through the LBL method. NF membranes incorporating PDADMAC as a polycation and PSS as a polyanion have demonstrated high efficiency in the removal of scale-forming divalent cations (Mg^2+^, Ca^2+^, Sr^2+^, and Ba^2+^) from feedwaters across a range of salinities. [16]. Additionally, LBL assemblies of poly[(N,N′-dicarboxymethyl) allylamine] (PDCMAA) and PAH have been reported for Cu^2+^/Mg^2+^ separation, showcasing promising results [17]. Remarkable performance has been observed in NF polyelectrolyte membranes with PEMs composed of PAH and PSS, contributing to an enhanced water softening performance for feed water [18]. Furthermore, these membranes have exhibited notable capabilities in Mg^2+^/Li^+^ separation [11,12]. These findings collectively highlight the versatility and potential of ion separation techniques, particularly in those utilizing NF membranes and LBL modifications, in addressing diverse separation challenges. In this study, we modified PAN hollow fibers through varying durations of hydrolyzation, employing the LBL deposition method with PAH and PSS up to 5 and 10 bilayers. The performance of these membranes was evaluated through Mg^2+^/Li^+^ separation using single salt (LiCl and MgCl_2_) solutions. The efficacy of the LBL nanofiltration hollow fiber membrane technique was confirmed by comparing the results with those of other studies, attesting to its superior performance.

## 2. Materials and Methods

### 2.1. Materials

PAN hollow fiber membranes were provided by Eco-Task Group Ltd., Hong Kong with inner/outer diameters of 1.1/2.1 cm and pore size 0.2 μm. Polycation electrolyte poly(allylamine hydrochloride) (PAH) was provided by Thermo Scientific Chemicals (Waltham, MA, USA). Polycation electrolytes, poly(diallyldimethylammonium chloride) (PDADMAC) and poly(ethyleneimine) (PEI, M_w_ ~ 750,000), were provided by Sigma Aldrich (St. Louis, MO, USA). Polyanion electrolyte poly(styrene sulfonate) (PSS, M_w_ = 70,000) was supplied from Thermo Scientific Chemicals (US). Glycerol was provided by Sordalab (Paris, France). Glutaraldehyde (GA 50% aqueous solution) and sodium hydroxide (NaOH) were provided by Fisher Scientific (Waltham, MA, USA). Magnesium chloride (MgCl_2_) and sodium chloride (NaCl) were supplied by Sigma Aldrich. Lithium chloride (LiCl) was supplied by Thermo Scientific Chemicals (US).

### 2.2. PAN Hollow Fiber Hydrolysis Treatment and Modification

PAN hollow fiber samples were first rinsed with deionized water. To start a hydrolysis process, the samples were subsequently immersed in a NaOH aqueous solution of 2 molar concentration for 1, 1.5, 2, and 2.5 h. The temperature of the hydrolysis reaction was maintained at 45 °C. Ultimately, each sample was removed, rinsed with deionized water, and allowed to air dry overnight. Hydrolyzed membranes without LBL deposition were labeled as HPAN.

**Figure 1:**
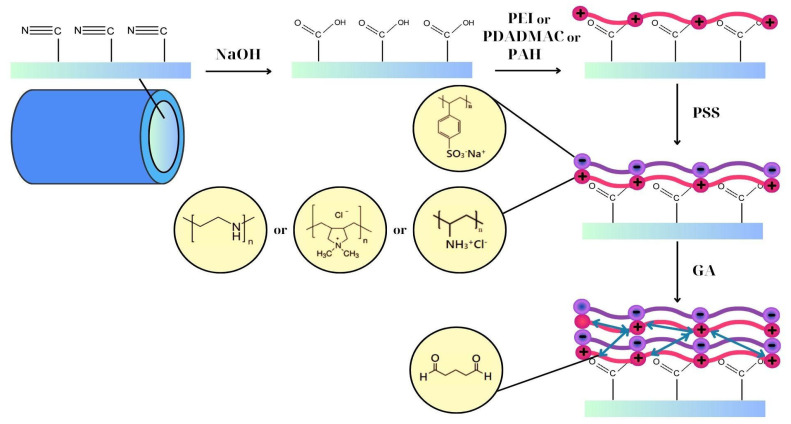
Illustration of LBL deposition on PAN hollow fibers and the chemical structures of polyelectrolytes and GA (light red and purple line layers represents positively and negatively charged polyelectrolytes respectively; double arrow green lines represent the cross-linker).

Three PAN hollow fibers with a length of 4 cm were used for LBL deposition and the hollow fiber area was 0.0003768 m^2^. Before coating, hollow fibers were rinsed with DI water at 2.0 bar for 10 min to remove the conservative chemicals and clean the inner surface. Glycerol solution 5% *w*/*w* was pumped into the fibers for 20 min, using a continuous flow pump provided by Automation & Power (Egaleo, Athens, Greece). The polycationic PAH solution was prepared using 1% *w*/*w* PAH and 15% *w*/*w* NaCl (final pH of the solution was 3.9). The polycationic PDADMAC solution was prepared using 5% *w*/*w* PDADMAC and 15% *w*/*w* NaCl (final pH of the solution was 7.2). The polycationic PEI solution was prepared using 2% *w*/*w* PEI and 15% *w*/*w* NaCl (final pH of the solution was 10.7). The polyanionic PSS solution was prepared using 0.4% *w*/*w* PSS and 15% *w*/*w* NaCl (final pH of the solution was 6.5). For the surface modification (LBL) of the HPAN hollow fibers, one type of polycationic solution was used each time with the polyanionic solution (PSS). Therefore, we developed three LBL combinations: (a) PAH/PSS, (b) PDADMAC/PSS, and (c) PEI/PSS. The polyelectrolyte solutions were pumped at 2.0 bar pressure for 5 min. Any excess of polyelectrolyte solutions was washed off by deionized water, at least two times. The number of bilayers produced in this study was five and ten for all three polyelectrolyte combinations. For example, one bilayer was produced by using one layer of PAH solution, followed by deionized water rinsing, and then with one layer of PSS solution, followed by deionized water rinsing. The resulting membranes were cross-linked by GA solution (1% *w*/*w*) for 15 min at room temperature 25 degrees Celsius. Polyelectrolyte multilayer hollow fibers were labeled as (PAH/PSS)_5_-X and (PAH/PSS)_10_-X, for 5 and 10 bilayers respectively, where X is the time of pretreatment. The deposition of polyelectrolytes on the membrane is illustrated in Figure 1. An example of PAN hollow fiber sample and an experimental set-up of LBL deposition are shown in Figure S1 and Figure S2 respectively on the supporting information (supplementary materials).

### 2.3. Characterization and Performance Evaluation Methods

**Figure 2:**
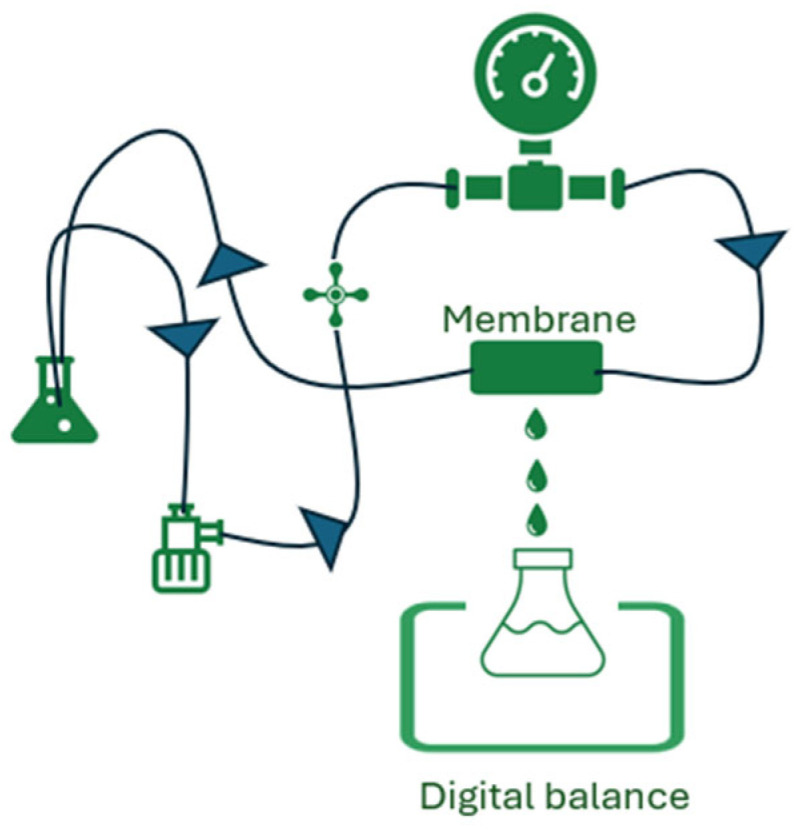
Experimental set-up for testing the metal ion concentration through the membrane (permeance).

The membrane morphology was characterized by means of scanning electron microscopy. The preparation consisted of quenching the samples in liquid nitrogen to ensure brittle fracture, and then the samples were coated with gold using a POLARON E5100 sputter coater provided by Polaron Equipment Ltd (Hertfordshire, England). A FEI Quanta Inspect scanning electron microscope (SEM) was used, in secondary electron mode, operating at 15 kV. Various magnifications and spot sizes (1.5–5) were selected to ensure the optimal imaging and chemical analysis conditions. For the chemical analysis, the working distance was set to 10 mm, and an energy-dispersive X-ray spectroscopy (EDS) super ultra-thin window detector by EDAX was used. Fourier transform infrared spectroscopy (FTIR) was performed using a Nicolet iS50 (Thermo Scientific) instrument to determine the chemical composition and modifications of PAN samples. The rejection, R, was determined through conductivity measurements conducted on both permeate and feed solutions (1000 mg/L) of MgCl_2_ (pH = 8) and LiCl (pH = 8.4). The solutions were pumped into the fibers at a pressure of 0.5–2 bar and the results were identified using a conductivity meter provided by Bante Instruments TDS-120W Conductivity Meter (Shanghai, China). Permeance, P (LMH/bar), was calculated as the volume of permeate water (L) per unit time (H) and surface area (M) under a unit applied pressure (bar) (Figure 2).

## 3. Results

### 3.1. Characterization of LBL Modified Hollow Fiber Membranes

**Figure 3:**
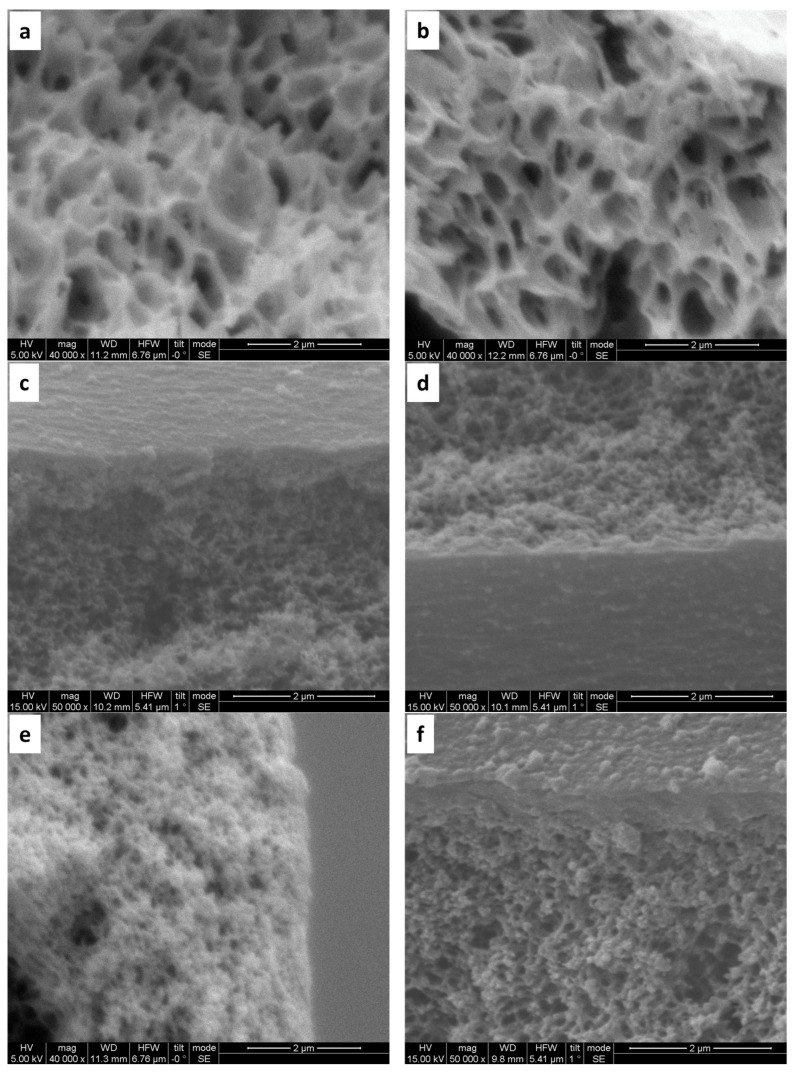
SEM images of PAN hollow fiber substrates: (**a**) unmodified substrate, exhibiting an average pore size of 200 nm (±50 nm); (**b**) modified substrates after a 2 h pretreatment with 2M NaOH; (**c**,**d**) modified with 2 h pretreatment with NaOH 2M and subsequent deposition of five bilayers of (PAH/PSS); (**e**,**f**) modified substrates after a 2 h pretreatment with NaOH 2M and subsequent deposition of 10 bilayers (PAH/PSS).

**Figure 4:**
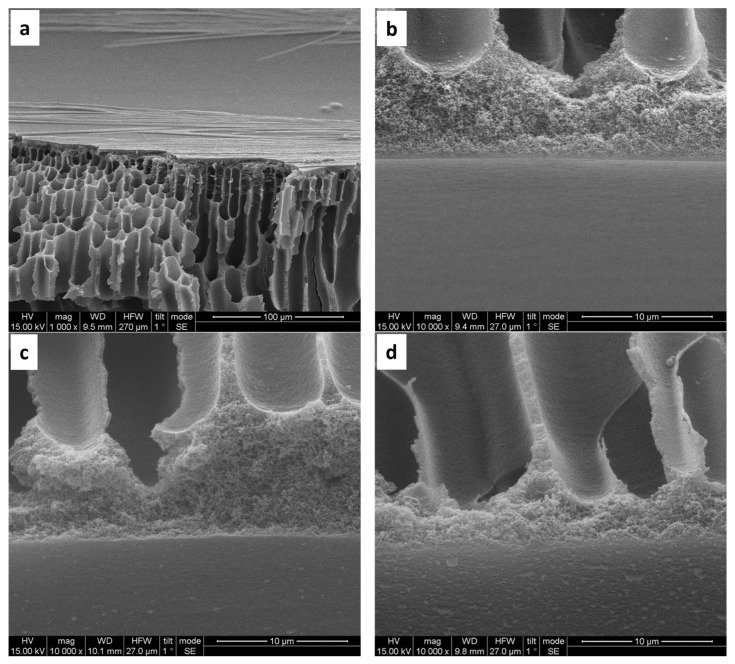
SEM images of PAN hollow fiber substrates: (**a**,**b**) unmodified substrate, exhibiting an average pore size of 200 nm (±50 nm); (**c**) modified with 30 min pretreatment with NaOH 2M and subsequent deposition of five bilayers of (PAH/PSS); (**d**) modified substrates after a 30 min pretreatment with NaOH 2M and subsequent deposition of 10 bilayers (PAH/PSS).

The inner surface of bare PAN hollow fibers displays small pores and a sponge-like pore structure (Figure 3a). Following hydrolysis, the structural morphology remains largely unchanged (Figure 3b). However, subsequent coating with (PAH/PSS) results in a noticeable reduction in surface pore size, observed with the formation of both 5 and 10 bilayers (Figure 3c–f). Microscopic examination of the side inner surface of bare PAN hollow fibers reveals a smooth morphology (Figure 4a,b). In contrast, the deposition of 5 bilayers (PAH/PSS) (Figure 4c), and to a greater extent, 10 bilayers, results in a progressively rougher surface texture (Figure 4d).

**Figure 5:**
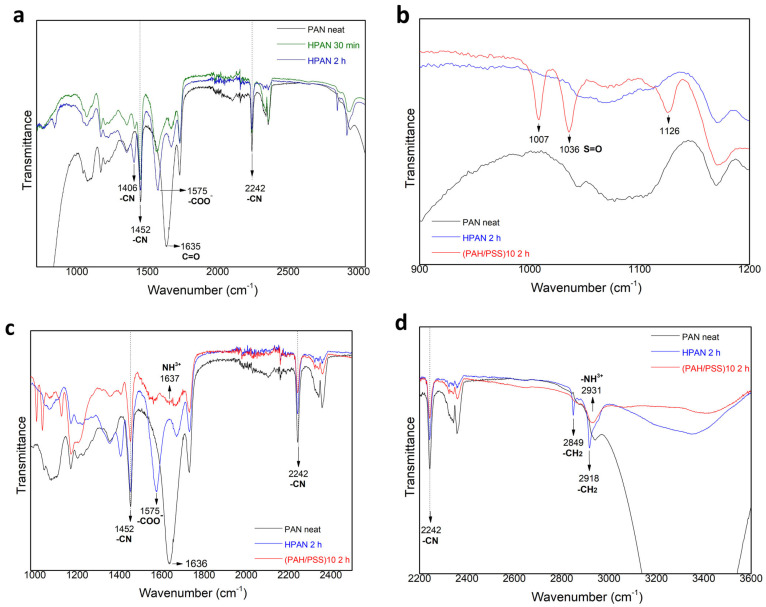
FTIR spectra of PAN hollow fiber with different pretreatment and (PAH/PSS) modification (**a**) PAN hollow fiber without pretreatment or modification, with 30 min and 2 h 2M NaOH pretreatment; (**b**–**d**) PAN hollow fiber without pretreatment or modification, with 2 h 2M NaOH pretreatment and 10 bilayer (PAH/PSS) deposition.

Figure 5a shows the transmittance FT-IR spectra of untreated and hydrolyzed PAN hollow fibers. The peak at 1635 cm^−1^ corresponds to the carbonyl group (C=O) of the untreated PAN sample. The peaks at 1452 and 2242 cm^−1^ belong to the C–N stretching of the –C≡N group. These bands significantly decrease in the hydrolyzed samples. Apparently, the peak intensity at 2242 cm^−1^ did not substantially change after hydrolysis, indicating that only a small number of nitrile groups (–C≡N) in untreated PAN hollow fiber were converted to the –C=N– groups [19,20]. A new peak at 1575 cm^−1^ is observed, which is assigned to amide (–CONH_2_) moieties [20]. Additionally, another new peak at 1406 cm^−1^ corresponds to imine groups (–C=N–) in the hydrolyzed samples [20], with the peak intensity decreasing for shorter pretreatment times. The presence of PSS in LBL modified samples is confirmed by three new peaks within 1000–1126 cm^−1^ wavelength (Figure 5b). The peaks at 1036 and 1126 cm^−1^ belong to symmetric stretching of the S-O [15,21]. The peak at 1637 cm−1 (Figure 5c) is attributed to protonated NH^3+^ groups in PAH-Cl while the peak at 2931 cm^−1^ corresponds to the stretching vibration of -NH_3_^+^ [15,21]. According to other studies, the peak at 2931 cm^−1^ (Figure 5d) may be attributed to the asymmetric vibration of the –CH_2_ groups of PAH-Cl [15].

### 3.2. Separation Performance for Mg^2+^ and Li^+^

**Figure 6:**
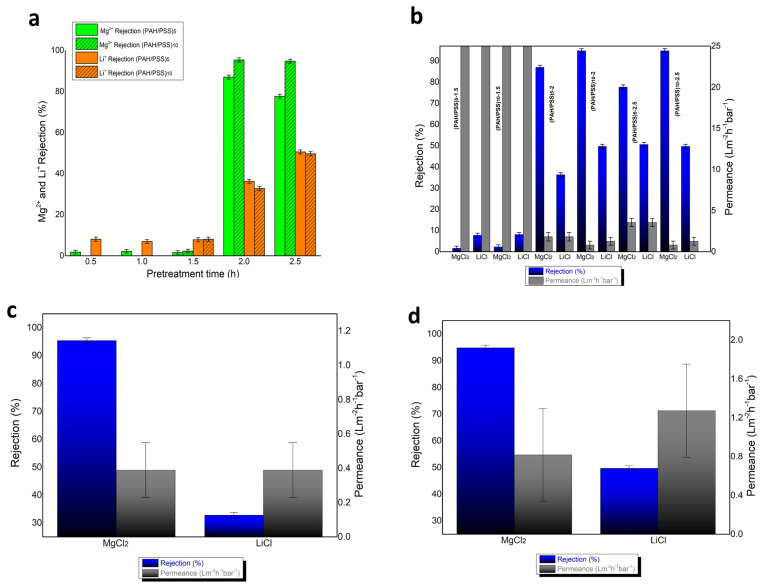
(**a**) Mg^2+^ rejection and Li^+^ rejection of all membranes with (PAH/PSS) deposition (**b**) Mg^2+^ rejection, Li^+^ rejection and permeance of PAN hollow fibers with 1.5–2.5 h time of hydrolysis and 5, 10 bilayers of (PAH/PSS) (**c**) Mg^2+^ rejection, Li^+^ rejection and permeance of (PAH/PSS)_10_-2 membrane (**d**) Mg^2+^ rejection, Li^+^ rejection and permeance of (PAH/PSS)_10_-2.5 membrane.

The rejection results of Mg^2+^ and Li^+^ appear to be significantly influenced by the hydrolysis time of PAN hollow fibers, with membranes exhibiting better rejection of Mg^2+^ and Li^+^ as the hydrolysis time increases, particularly beyond 2 h (Figure 6a). Simultaneously, it is observed that, for hydrolysis times less than 1.5 h, there is no significant change in the rejection of Mg^2+^ and Li^+^ (Figure 6a). It seems that the carboxylic groups formed on the surface of the hydrolyzed PAN hollow fibers acted as functional sites for the deposition of the PAH layer. Additionally, Mg^2+^ rejections increase with the number of PAH/PSS bilayers, while Li^+^ rejections remain relatively stable. The factors affecting the separation of Mg^2+^ and Li^+^ include the pore size of the membrane, as well as the Donnan exclusion. In nanofiltration membranes, pore size acts as a key steric hindrance, meaning larger ions like Mg^2+^, which have a larger hydrated radius (0.43 nm), are more restricted in passing through the pores compared to smaller ions like Li^+^ (0.38 nm) [12]. In this study, the addition of layers through LBL deposition minimizes the pore size of the membranes, further enhancing steric hindrance for Mg^2+^ ions. This reduction in pore size directly impacts the membrane’s selectivity, as the smaller pores created by LBL deposition restrict the passage of larger ions more effectively, resulting in improved separation performance between Mg^2+^ and Li^+^. Moreover, due to its single positive charge, Li^+^ likely exhibits reduced rejection as a result of limited Donnan exclusion. It appears that Mg^2+^ ions, having a higher charge density than Li^+^ ions, may interact more intensely with the sulfonic groups of PSS, potentially leading to preferential penetration or retention of one ion over the other. Permeance drastically decreases for membranes with hydrolysis times of 2 and 2.5 h, as well as for membranes with a higher number of PAH/PSS bilayers (Figure 6b). This effect may be attributed either to hydrolysis or to a reduction in the pore size of the membrane. Membranes (PAH/PSS)_10_-2 (Figure 6c) and (PAH/PSS)_10_-2.5 demonstrate nearly perfect rejections of MgCl_2_ (95.4 and 94.8 respectively) and similar rejections of LiCl (32.8 and 49.7 respectively). The membrane (PAH/PSS)_10_-2.5 exhibited optimal results in terms of rejection efficiency and permeability and is characterized by permeability of 0.81 and 1.27, respectively, for MgCl_2_ and LiCl (Figure 6d). To evaluate the long-term stability of the fabricated membranes, a stability assessment was conducted on the two highest-performing membranes. This assessment demonstrated consistent performance in both MgCl_2_ and LiCl rejection and permeance across three distinct operational cycles, with measurements taken weekly over a three-week period. The results are presented in Table 1.

**Figure 7:**
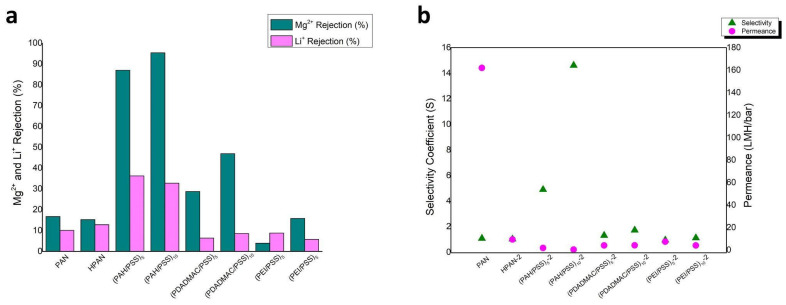
(**a**) Comparison of the performance of PAH/PSS, PDADMAC/PSS and PEI/PSS LBL membranes. (**b**) comparison of the selectivity coefficient and permeance of PAH/PSS, PDADMAC/PSS and PEI/PSS LBL membranes.

Upon examining the effects of hydrolysis duration on membrane performance, the pretreatment condition of 2 h with 2M NaOH solution was identified as the most effective. This pretreatment was used to evaluate the performance of the membranes when modified with polyelectrolytes PEI and PDADMAC. Analytical results demonstrate that membranes modified with PAH exhibit superior separation efficiency compared to those modified with PEI and PDADMAC (Figure 7). Furthermore, an increase in the number of bilayers correlates with an enhanced solute rejection rate, accompanied by a corresponding decrease in permeance, indicating a trade-off between separation efficiency and membrane permeability. The rejection, selectivity coefficient, and permeance values of the PAN hollow fibers are listed in Table 2. The selectivity coefficient (S) was calculated as:

S = (1 − R_Li)/(1 − R_Mg)

where R_Li and R_Mg are the rejection rates for Li^+^ and Mg^2+^, respectively.

## 4. Discussion

Comparing the (PAH/PSS)_10_-2 and (PAH/PSS)_10_-2.5 membranes with those reported in the literature, the results are similarly advantageous. LBL PES hollow fiber NF membranes assembled by (PSS/PAH) demonstrated high rejection rates for Mg^2+^ (99.1%) and Li^+^ (59.4%), and were characterized by a water permeability of 18.4 Lm^−2^h^−1^bar^−1^ [12]. Additional research on (PSS/PAH) PES hollow fibers, both with and without crosslinking, exhibited very high (>90%) rejections of MgCl_2_ and a comparable rejection rate (~80%) for LiCl [11]. Similar ranges of MgCl_2_ rejection rates (96–98%) were observed with (PSS/PAH)_2.5_ membranes, with slightly lower values (91–97%) for the corresponding crosslinked membranes [13]. Particularly for Mg^2+^, further studies have been conducted, such as those involving PES hollow fibers deposited with only two bilayers of (PSS/PAH) polyelectrolytes [22] and crosslinked PES hollow fibers with (PSS/PAH) layers [18]. These studies exhibited a Mg^2+^ rejection rate of 94%, with a water permeability of around 12 LMH/bar and a rejection rate over 95% of MgCl_2_ with more than 10 LMH/bar permeability, respectively.

The current study achieved a Mg^2+^ rejection rate of 95.4% and a Li^+^ rejection rate of 32.8% with ten bilayers of (PAH/PSS) on hydrolyzed PAN fibers. These results are compared with other studies on LBL modified membranes (Table 3). The performance aligns with findings by He et al., who reported Mg^2+^ rejection rates of 96–98% using (PSS/PAH)_2.5_ membranes [11]. Similarly, another study demonstrated a Mg^2+^ rejection rate of over 95% using LΒL-modified PES membranes [18]. These comparisons underscore the robustness of the LΒL technique in achieving high Mg^2+^ rejection rates consistently across different membrane substrates and configurations. The permeability observed in the current study (0.39 LMH/bar) for the optimized (PAH/PSS)_10_-2 membrane is lower compared to some reports. The lower permeability might be due to the smaller pore sizes resulting from extended hydrolysis times and multiple bilayers, indicating a trade-off between rejection efficiency and permeability. Nevertheless, the extended hydrolysis time was essential to provide smaller membrane pore size, but also negatively charged -COO^−^ groups on the hydrolyzed PAN surface, and thus assisting in the deposition of positively charged PAH layers. The findings of Bruening et al. present two distinct approaches utilizing polyelectrolytes PAH/PSS for Mg^2+^/Li^+^ separation. In one approach, Nafion membranes were coated with alternating layers of PAH and PSS. This LBL approach created a highly selective barrier that favors the transport of Li^+^ while effectively rejecting Mg^2+^ due to size and charge interactions. The polyelectrolyte multilayers created an environment where smaller monovalent ions like Li^+^ could pass through more easily than larger divalent ions like Mg^2+^. This was due to both steric hindrance and electrostatic repulsion, as the fixed charges in the membrane repel similarly charged co-ions [23]. Additionally, in another study, Nafion membranes were also coated with alternating layers of PAH and PSS. This LBL approach enhanced the selectivity for monovalent ions like Li^+^ over divalent ions like Mg^2+^. The pH of the solution played a critical role in the performance of these membranes. Increasing the pH from 6.5 to 8.3 significantly enhanced the cation fluxes and limiting currents, making the membrane more cation-permselective. At higher pH levels, the selectivity for Li^+^ over Mg^2+^ increased dramatically, achieving a selectivity ratio greater than 700 at neutral pH. This was attributed to the enhanced permeability of monovalent cations through the polyelectrolyte-coated membranes. The proposed mechanism for separating lithium from magnesium involved a combination of selective polyelectrolyte multilayer coatings on ion-exchange membranes, optimized pH conditions, and effective ion transport dynamics under an electric field. This innovative approach offers high selectivity and recovery rates, making it a promising method for lithium extraction in various industrial applications [24]. Notably, the literature indicates that GA crosslinking in LBL NF membranes effectively reduces pore size, thereby enhancing size-based exclusion effects. Additionally, GA crosslinking reduces positive charge density by transforming amine groups into neutral imine bonds, which minimizes the membrane’s attraction to smaller ions. This modification further improves Mg^2+^/Li^+^ selectivity by favoring the rejection of larger, multivalent ions while reducing permeability to smaller monovalent ions [11,12,13]. In a different study, PAH, PEI, PDADMAC as positively charged polyelectrolytes were deposited with PSS on PES membranes [25]. It was found that the Mg^2+^ rejection was higher than 95% when PAH was used and the Mg^2+^ rejection was decreasing in the order of using positively charged polyelectrolytes as PAH > PDADMAC > PEI. This result is in accordance with our experimental results where it was found that (PAH/PSS) had the best Mg^2+^ rejection followed by (PDADMAC/PSS) and then (PEI/PSS). An explanation for this is probably due to the molecular structure of PAH, where the NH_3_^+^ is found to be perpendicular to the polymer chain, and thus readily available to interact with the negatively charged PSS, without any hindrance effect. On the other side, the positive ions of PDADMAC (N^+^) are surrounded by methyl groups, and thus their positively charged groups are hindered. Similarly, the positive ions of PEI are found in the main back-bone polymer chain and thus hindered from the interaction with negatively charged PSS ions. In comparison to the study by Rajesh et al. [26], which explored the use of ionically crosslinked polyelectrolyte multilayers of BPEI and polyacrylic acid (PAA) on PAN nanofiber mats modified with cellulose acetate for the separation of ionic impurities, our current study using PAN hollow fiber membranes modified with PAH/PSS bilayers demonstrates several key similarities and differences. Both studies employ the LΒL assembly technique to enhance ion separation efficiency. Rajesh et al. achieved a MgSO_4_ rejection of 98.7% with a water flux of 19.7 L/m^2^/h using 15 bilayers of BPEI/PAA, indicating a highly effective separation layer with significant flux capabilities [26]. In contrast, our study achieved a Mg^2+^ rejection rate of 95.4% using 10 bilayers of PAH/PSS, highlighting the efficacy of PAH/PSS in achieving high rejection rates with fewer bilayers compared to BPEI/PAA. A notable difference lies in the ion separation capabilities of lithium. Rajesh et al. did not specifically address Li^+^ separation, focusing primarily on multivalent ions like Mg^2+^ and SO_4_^2−^ [26]. Our study, however, achieved a Li^+^ rejection rate of 34.8%, underscoring the selective separation challenges posed by monovalent ions like lithium. The lower permeability in our study (0.39 LMH/bar) compared to Rajesh et al. (19.7 LMH/bar) may be attributed to the smaller pore sizes resulting from extended hydrolysis times and multiple bilayers, indicating a trade-off between rejection efficiency and permeability. The use of different polycations also plays a critical role in separation efficiency. Rajesh et al.’s use of BPEI, known for its high charge density and amine group availability, contrasts with our use of PAH, PDADMAC and PEI, which similarly provides effective charge interaction, but with a different molecular structure and steric effects. Another reason is that Rajesh et al. did not hydrolyze PAN fiber mats but applied a cellulose acetate layer using spin-cast. The work of Wang et al. with B15C5-MX-NF on PES showed a relatively good separation performance [3].

Considering the successful outcomes and comparisons with existing research, future studies could explore optimizing the LBL assembly process to balance permeability and rejection rates with the use of other polyelectrolytes. Additionally, investigating the long-term stability and fouling resistance of these membranes in real-world applications would provide valuable insights for scaling up the technology for industrial use.

By considering a preliminary techno-economic study (without providing many details in this research work), the (PAH/PSS)_5_-2 membrane could offer a superior balance of cost-effectiveness, permeance, and Mg^2+^/Li^+^ selectivity, making it a better choice for most industrial applications focused on lithium extraction from brine. Its higher permeance could reduce both capital and operating costs, making it more feasible for large-scale operations.

The (PAH/PSS)_10_-2 membrane, while providing higher Mg^2+^ rejection and better selectivity, could incur higher costs due to its much lower permeance. This membrane could be more suitable for niche applications, where maximum Mg^2+^ separation is essential despite the increased capital and operational expenditures.

## 5. Conclusions

The study explored the separation of magnesium and lithium ions using PAN hollow fiber membranes modified through an LBL polyelectrolyte technique. The LBL-modified PAN hollow fiber membranes demonstrated significant improvements in the separation efficiency of Mg^2+^ and Li^+^ ions. The study found that ten bilayers of PAH/PSS on hydrolyzed PAN fibers resulted in a rejection rate of 95.4% for Mg^2+^ and 34.8% for Li^+^ ions. This highlighted the method’s effectiveness in selectively targeting and separating specific ions based on their charge and size. The hydrolysis time of the PAN hollow fibers was a crucial factor in enhancing the ion rejection rates. Membranes hydrolyzed for more than 2 h showed better performance in rejecting Mg^2+^ and Li^+^ ions. The increased hydrolysis time facilitated the formation of carboxylic groups, which acted as functional sites for polyelectrolyte deposition. Among the polycations tested, PAH was identified as the most effective in enhancing Mg^2+^ rejection. This superior performance was attributed to the molecular structure of PAH, where NH_3_^+^ groups were readily available for interaction with negatively charged PSS without significant hindrance. In contrast, other polycations like PDADMAC and PEI showed lower Mg^2+^ rejection due to steric hindrance from the surrounding groups. This study noted that the permeance of the membranes decreased with longer hydrolysis times and increased bilayer numbers. This was likely due to a reduction in pore size, which provides greater hindrance to ion passage. The optimized (PAH/PSS)_10_ membrane exhibited an optimal balance between rejection efficiency and permeability, highlighting the importance of controlling the LBL assembly process to achieve desired performance. The findings from this study were consistent with previous research on LBL-modified NF membranes. The results confirmed that PAH/PSS-modified membranes offer superior Mg^2+^ rejection compared to other polyelectrolytes. The study’s outcomes align with the reported high rejection rates and permeability values from similar LBL nanofiltration membrane studies, underscoring the technique’s robustness and applicability in ion separation processes.

Overall, the research demonstrates that the LBL modification of PAN hollow fibers is a promising and effective method for the selective separation of Mg^2+^ and Li^+^ ions, with potential applications in lithium extraction from brines and lithium battery recycling. To further address the issue of low permeance, future studies could explore how combining different polyelectrolytes (for example 5 to 10 bilayers of PDADMAC/PSS and 2 to 4 bilayers of PAH/PSS), increase the number of bilayers that showed good permeance to 20 bilayers, or introduce intermediate layers such as graphene oxide to those bilayers to enhance both separation efficiency and permeance. Finally, to address the issue of low permeance, future studies could include the use of a smaller number of bilayers on PAN membranes with less than 200 nm pore size, the use of ion solutions at different pHs, and the use of electrodes and applied electric fields that could enhance both selectivity and permeance.

## Figures and Tables

**Table 1:** Summary of the performance of (PAH/PSS)_10_-2 and (PAH/PSS)_10_-2 membranes after three weekly measurements.

	1st Week		2nd Week		3rd Week	
	(PAH/PSS)_10_-2	(PAH/PSS)_10_-2.5	(PAH/PSS)_10_-2	(PAH/PSS)_10_-2.5	(PAH/PSS)_10_-2	(PAH/PSS)_10_-2.5
Mg Rejection (%)	95.40 ± 0.9	94.8 ± 1	96.00 ± 0.9	94.5.2 ± 1	95.36 ± 0.9	94.8 ± 1
Li Rejection (%)	32.80 ± 0.9	49.7 ± 1	32.80 ± 0.9	49.6 ± 1	31.90 ± 0.9	49.8 ± 1
Permeance (LMH/bar)	0.39 ± 0.47	1.04 ± 0.52	0.34 ± 0.47	1.05 ± 0.52	0.35 ± 0.47	1.05 ± 0.52

**Table 2:** Summary of the performance of hydrolyzed and modified PAN membranes.

Sample Name	Mg Rejection (%)	Li Rejection (%)	Selectivity Coefficient (S)	Permeance (LMH/bar)
PAN	16.73 ± 1	10.11 ± 1	1.079	161.91 ± 0.51
HPAN-2	15.30 ± 1.4	12.80 ± 1.4	1.030	9.37 ± 0.5
(PAH/PSS)_5_-2	87.00 ± 1	36.30 ± 1	4.900	1.83 ± 0.49
(PAH/PSS)_10_-2	95.40 ± 0.9	32.80 ± 0.9	14.609	0.39 ± 0.47
(PDADMAC/PSS)_5_-2	28.74 ± 1	6.39 ± 1	1.313	4.07 ± 0.49
(PDADMAC/PSS)_10_-2	46.95 ± 1	8.53 ± 1	1.724	4.11 ± 0.49
(PEI/PSS)_5_-2	3.97 ± 1.1	8.83 ± 1.1	0.949	7.46 ± 0.5
(PEI/PSS)_10_-2	15.83 ± 0.9	5.79 ± 0.9	1.119	4.03 ± 0.48

**Table 3:** Comparison of the current results with other previous works on LBL modified membranes for monovalent/divalent ion separation.

Study	Membrane Type	Mg^2+^ Rejection (%)	Li^+^ Rejection (%)	Selectivity Coefficient	Permeance (LMH/bar)	Notes
Current Study	(PAH/PSS)_10_ on hydrolyzed PAN hollow fiber	95.4	32.8	14.61	0.39	High/average selectivity coefficient. Low permeance.
Current Study	(PAH/PSS)_5_ on hydrolyzed PAN hollow fiber	87.00	36.30	4.900	1.83	Average selectivity coefficient. Low/average permeance.
[12]	(PSS/PAH)_2.5_ on PES hollow fiber	99.1	59.4	45.11	18.4	High selectivity coefficient. High permeance.
[18]	Crosslinked (PSS/PAH)1.5C on PES hollow fiber	98.2	33.5 (Na^+^)	36.94	10	High selectivity coefficient. High permeance.
[18]	(PSS/PAH)_2_ on PES hollow fiber	98.1	58.6 (Na^+^)	21.79	>10	High/average selectivity coefficient. High permeance.
[18]	NF 270	42.9	37.1 (Na^+^)	1.10	>10	Commercial membrane, low selectivity coefficient. High permeance.
[18]	NF 90	96	68.6 (Na^+^)	7.85	6–7	Commercial membrane, average selectivity coefficient. High/average permeance.
[26]	(BPEI/PAA)_15_ on PAN nanofiber mats coated with CA	95.3	34.1 (Na^+^)	14.02	5	Different polyelectrolytes, average selectivity coefficient. High/average permeance.
[3]	B15C5-MX-NF on PES	89.7	21.4	7.63	10.8	Different polyelectrolytes, average selectivity coefficient. High permeance.

## Data Availability

The original contributions presented in this study are included in the article and Supplementary Material. Further inquiries can be directed to the corresponding authors.

## Supplementary Materials

The following supporting information can be downloaded at: https://www.mdpi.com/article/10.3390/ma17235878/s1, Figure S1: Pan hollow fiber sample; Figure S2: Experimental set-up of LBL deposition.
