# Supplementary material for: Separation of Magnesium and Lithium Ions Utilizing Layer-by-Layer Polyelectrolyte Modification of Polyacrylonitrile Hollow Fiber Porous Membranes
Source: Materials (Basel). 2024 Nov 30;17(23):5878. doi: 10.3390/ma17235878 (PMC11642634; doi:10.3390/ma17235878)
Supplement: Supplementary file 1 [file materials-17-05878-s001.zip › materials-3331121-supplementary.pdf]

materials

Supplementary Materials

Separation of Magnesium and Lithium Ions Utilizing Layer-by-Layer Polyelectrolyte Modification of Polyacrylonitrile Hollow Fiber Porous Membranes

Danai Koukoufilippou [1], Ioannis L. Liakos [1,*], George I. Pilatos [1], Niki Plakantonaki [1], Alexandros Banis [1] and Nikolaos K. Kanellopoulos [1,2,*]

[1] Institute of Nanoscience and Nanotechnology, National Centre for Scientific Research Demokritos, Patr. Gregoriou E & 27 Neapoleos Street, 15341 Agia Paraskevi, Greece; danaikoukoufilippou@gmail.com (D.K.); g.pilatos@inn.demokritos.gr (G.I.P.); n.plakantonaki@inn.demokritos.gr (N.P.); a.banis@inn.demokritos.gr (A.B.)

[2] High Technology Filters (HTF) S.A., Siokou Street 18, 15341 Agia Paraskevi, Greece

* Correspondence: i.liakos@inn.demokritos.gr (I.L.L.); n.kanellopoulos@inn.demokritos.gr (N.K.K.)

Supporting Information

Three PAN hollow fibers, each 4 cm in length, were used for layer-by-layer deposition. The PAN fibers were bonded between two holders using Huntsman Araldite 2000 adhesive, allowing the solutions to flow through the interior of the fibers to facilitate the deposition of the layers (Figure S1).

All the solutions were pumped into the fibers, using a continuous flow pump. The experimental set-up is shown in Figure S2. The parameters of the layer-by-layer deposition process, as well as those for testing the metal ion concentration, are as follows: Pressure 1.5 bar, Temperature 25 °C, pump voltage 3.2 V, pump current 0.5 A, deposition time per layer 5 min. Flow rate 0.1 LPM (litre per minute).

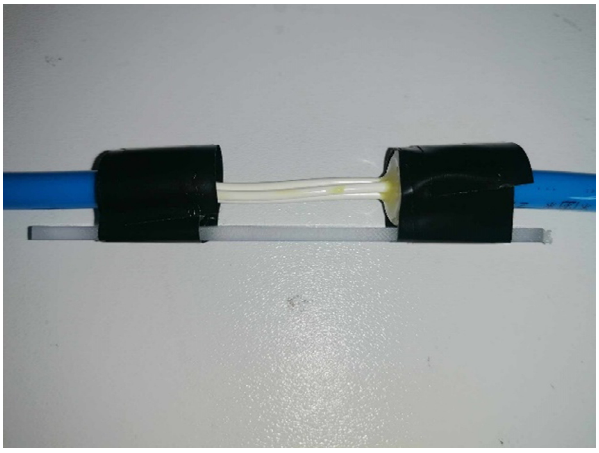

Figure S1. PAN hollow fiber sample.

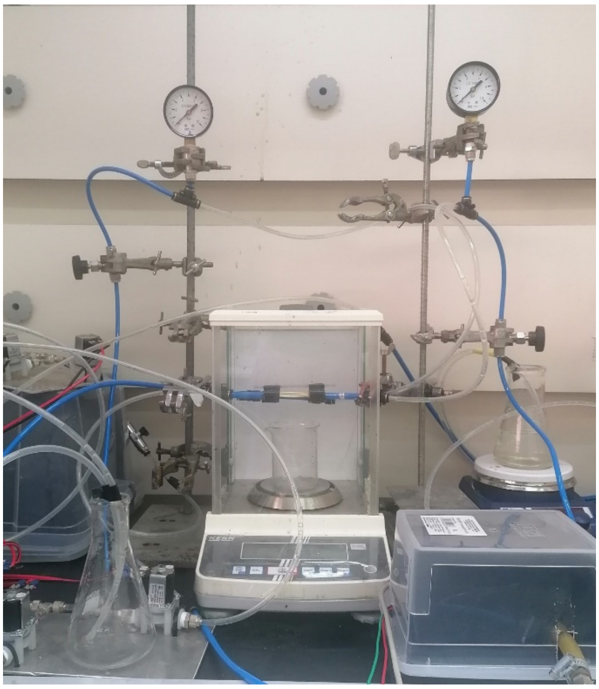

Figure S2. Experimental set-up of layer-by-layer deposition.
